# Clinical Differences between Men and Women with Psoriatic Arthritis: Relevance of the Analysis of Genes and Polymorphisms in the Major Histocompatibility Complex Region and of the Age at Onset of Psoriasis

**DOI:** 10.1155/2013/482691

**Published:** 2013-04-16

**Authors:** Rubén Queiro, Patricia Tejón, Pablo Coto, Sara Alonso, Mercedes Alperi, Cristina Sarasqueta, Segundo González, Jesús Martínez-Borra, Carlos López-Larrea, Javier Ballina

**Affiliations:** ^1^Rheumatology Department, Hospital Universitario Central de Asturias (HUCA), C/Celestino Villamil s/n, Oviedo, 33006 Asturias, Spain; ^2^Psoriasis Unit, Hospital Universitario Central de Asturias (HUCA), Oviedo, Asturias, Spain; ^3^Dermatology Department, Hospital Universitario Central de Asturias (HUCA), Oviedo, Asturias, Spain; ^4^Clinical Epidemiology Unit, BIODONOSTIA, San Sebastian, Basque Country, Spain; ^5^Department of Functional Biology, Oviedo University School of Medicine, Oviedo, Asturias, Spain; ^6^Immunology Service, HUCA, Oviedo, Asturias, Spain

## Abstract

It has been shown that males with spondyloarthritis tend to suffer from more severe spinal disease while females are more likely to have peripheral joint involvement. Nevertheless, gender-related differences have not been thoroughly explored in psoriatic arthritis (PsA). In PsA, males accumulate more peripheral and axial joint damage compared to women. However, it is not clear whether these findings are secondary to differences in occupational physical activity, hormonal changes, or other factors. The present study analyzed the differences in clinical expression of PsA between men and women. We have also evaluated the possible existence of gender-linked differences in the distribution of genes and polymorphisms within the major histocompatibility complex and whether patients' age at the onset of psoriasis established any differences in these aspects. Women suffered more polyarthritis, greater functional impairment, and a larger number of swollen joints during followup. We appreciated a differential expression of certain MHC genes according to gender and age at onset of psoriasis. Our results point to the need to include patient's age at the onset of psoriasis and gender as key stratification elements in future studies of genetic associations in PsA.

## 1. Introduction

Psoriasis is a relatively common T-cell mediated cutaneous disease in which both environmental and genetic factors contribute to its pathogenesis [1]. Despite multiple susceptibility loci for psoriasis that have been identified, the PSORS1 locus located in the MHC class I region on chromosome 6p21.3 confers the most risk for psoriasis [1]. However, the extensive ranges of polymorphism and the preservation of HLA haplotypes in most populations have made it difficult to characterize the exact gene responsible for psoriasis susceptibility located in PSORS1. Nevertheless, HLA-C*06 is the strongest association found in psoriasis and is the only genetic variant repeatedly observed to be associated with psoriasis in most of the populations (1). Additionally, other MHC genes have also been associated with psoriasis independently of HLA-C*06 [2, 3].

Psoriatic arthritis (PsA) is a chronic inflammatory joint disease which affects a significant proportion (10–40%) of psoriatic patients [4, 5]. HLA-C*06 has also been associated with PsA susceptibility (6). MICA-A9 (corresponding to MICA*002) polymorphism has been further associated with arthritis susceptibility but not with psoriasis [6–8]. Additionally, HLA-B38, HLA-B39, HLA-B57, HLA-DR7, and TNF-*α* promoter polymorphisms have been associated with PsA susceptibility in the past; however, these alleles are in linkage disequilibrium (LD) with HLA-C*06 or MICA-A9 [6–8]. Thus, it has been postulated that there are two different susceptibility loci associated with PsA in the MHC region: HLA-C*06, which is associated with the skin lesions (present in the extended haplotypes-EH-13.1, 37.1, and 57.1) and another, MICA-A9, associated with susceptibility to arthritis and present in EH38.1, EH39.1, and EH57.1. Therefore, haplotype EH57.1 (HLA-C*06-B57-DRB1*07-DQA1*02-DQB1*03) is associated with both psoriasis and inflammatory arthropathy.

It has been shown that males with spondyloarthritis tend to suffer from more severe spinal disease while females are more likely to have peripheral joint involvement [9]. Nevertheless, gender-related differences have not been thoroughly explored in PsA. In a recent investigation among patients with PsA, men tended to accumulate more peripheral and axial joint damage compared to women. However, it was unclear whether these findings were secondary to differences in occupational physical activity, hormonal changes, or other factors [10]. It is also plausible that some of these gender-observed differences in PsA could be attributed to genetic factors. Indeed, some studies have shown a close correlation between male sex, HLA-B27 positivity, and risk of psoriatic spondyloarthritis [11, 12]. Very few studies have investigated whether there are differences in the clinical and genetic risk profiles between men and women with PsA [13].

The present study analyzes the differences in clinical expression of PsA between men and women. We have also evaluated the possible existence of gender-linked differences in the distribution of genes and polymorphisms within the MHC and whether patients' age at the onset of psoriasis establishes any differences in these aspects.

## 2. Patients and Methods

### 2.1. Patients

One-hundred ten consecutive patients who fulfilled the Classification criteria for psoriatic arthritis (CASPAR) criteria [14] were randomly selected from the rheumatology outpatient clinic of a tertiary care hospital. There were 55 men and 55 women with a mean age of 48 ± 12 years. The mean disease duration for psoriasis was 19 ± 10 years and 13 ± 8 years for arthritis. Psoriasis preceded the arthritis in 75% of cases and a family history of psoriasis was recorded in 43% of patients. The joint patterns on the baseline visits were those described by Moll and Wright [15], though in the final analysis increased emphasis was placed on the dominant joint pattern over the last 5 years of followup. Accordingly, 39% had oligoarthritis, 29% polyarthritis, and 32% presented a predominantly axial disease. Distal interphalangeal joint disease was seen in 32% of patients, whereas nail disease was seen in 43.6%. Dactylitis was noted in 30% of patients. Clinical enthesitis was present in 30% of subjects. All the disease variables, including laboratory, clinical and exploratory tests, disease activity, physical function, joint counts, and radiological studies were investigated on a standard basis in both men and women. The population was stratified by age at the time of onset of psoriasis, adopting a cutoff point of 40 years for distinguishing between early and late psoriasis. The study was carried out with the approval of the ethics committee of our hospital. All patients gave written informed consent before enrolling in the study.

### 2.2. HLA Typing

Human leukocyte antigen, HLA-C, was typed using the polymerase chain reaction with sequence-specific primers (PCR-SSP) [7]. The polymorphisms of the octamer transcription factor 3 gene (OTF3), the corneodesmosin gene (CDSN), and the *a*-helix coiled-coil rod homologue (HCR) gene were analyzed as described previously [7]. The microsatellites C1_2_5 and C1_4_4 were amplified using the PCR primers described by Tamiya et al. [16]. HLA-B typing was carried out using the Dynal RELI SSO HLA-B test following the manufacturer's instructions. HLA-DRB1 alleles were typed and subtyped by performing polymerase chain reaction using specific primers (PCR-SSP). For the analysis of microsatellite repeat polymorphism in the MICA gene, PCR was carried out using primers labeled at the 5′ end with the fluorescent reagent Cy5 as previously described [7]. For MICB typing, a dinucleotide microsatellite polymorphism in the first intron was analyzed by PCR analysis and TNF-*α* polymorphism at positions −238 and −308 was typed by PCR as previously described [7]. The distribution of these markers was analyzed in the whole group as well as in both men and women separately. This distribution was also analyzed according to patients' age at the onset of psoriasis, using the previously mentioned cutoff point. All previous typing was also performed in a control population of 110 (55 men and 55 women) random blood donors matched ethnically and geographically.

### 2.3. Statistical Analysis

The differences between the frequencies of these allelic markers in patients and controls, as well as the differences found in accordance with sex and age at disease onset, were assessed by univariate analysis using a Fisher's exact test, chi-squared test with Yates' correction and a Student's *t*-test. The differences between genders corresponding to the categorical variables were assessed with Fisher's exact test, while Student's *t*-test was used in the case of the continuous variables. The odds ratio (OR) was calculated by the cross-product ratio and the 95% confidence intervals by the cornfield method. The *P* significant values were corrected (*P*_*c*_) by multiplying them by the number of alleles at each locus. The extent of linkage disequilibrium between two loci is expressed as the observed disequilibrium value (*λ*_s_), that is, a proportion of the theoretical maximum disequilibrium value (*λ*_max⁡_) achievable for this combination of alleles. The *λ*_s_ were calculated using the formula: *λ*_s_ = *λ*/*λ*_max⁡_ = Pab − (Pa · Pb)/Pa · (1 − Pb).

## 3. Result

### 3.1. Differences in the Clinical Variables of Disease between Men and Women

Compared to PsA males, females tended to be affected more frequently by polyarthritis as the main joint pattern during followup (40% versus 20%, *P* < 0.05), higher HAQ values (1.10 ± 0.53 versus 0.62 ± 0.42, *P* < 0.01), and higher swelling joint count (5.2 ± 4.6 versus 3.2 ± 4.2 *P* < 0.05). There were no gender differences in age at the onset of psoriasis or arthritis, family history of disease, distal interphalangeal involvement, dactylitis, nail disease, the presence of erosive disease in the radiological study, or severity of psoriasis (Table 1).

### 3.2. Differences in Disease Variables between Men and Women according to Age at the Onset of Psoriasis (Cutoff Point 40 Years)

Most patients developed psoriasis before 40 years of age (45 men and 40 women). These individuals showed a family history of psoriasis in 47 cases (40%), while the rest of the patients with psoriasis first manifesting after 40 years of age presented antecedents of the disease in only three cases (12%), *P* < 0.05. In general, the psoriasis-arthritis latency period was significantly shorter in patients with late psoriasis (3.5 ± 3.4 years) than in subjects with early psoriasis (7.3 ± 5.4 years) (*P* < 0.01). The only discordant data between genders in the patients with psoriasis developing before 40 years of age were a shorter psoriasis-arthritis latency period in men (5.5 ± 7.1 years) than in women (9.3 ± 6.6 years) (*P* < 0.01), and a greater frequency of polyarthritis during followup in women (37.5% versus 17.7%) (*P* < 0.05). Women with early psoriasis showed greater nail involvement than men, though the difference was not statistically significant. In women with type II psoriasis, polyarthritis likewise was the dominant PsA pattern (53.3% versus 30%), though the differences failed to reach statistical significance, due to the small number of cases in this group. In the same way, in men with type II psoriasis, the dominant joint pattern was axial involvement (70% versus 33.3%), though once again the differences failed to reach statistical significance, due to the small number of cases in this group (Table 2).

### 3.3. Comparative Distribution of MHC Genetic Markers between Men and Women

The global study population showed a relationship between the presence of HLA-C*06 (56.4% versus 17%, OR 6.18, 95% CI: 3.32–11.5, *P*_*c*_ < 0.0001), MICA-A9 (60% versus 30%, OR 3.5, 95% CI: 2.0–6.12, *P*_*c*_ < 0.001), and HLA-B*27 (31.8% versus 7.3%. OR 5.9, 95% CI: 2.6–13.4, *P*_*c*_ = 0.001) and the risk of suffering PsA. Subjects expressing HLA-C*06 were significantly younger at the time of onset of psoriasis (24 ± 13.6 years) than the individuals without this allele (32.4 ± 14.1 years), *P* = 0.01. With respect to HLA-C*06 and family history, 64% of subjects who showed a positive family history had this allele compared to 44% of those with no family history, OR 2.3, 95% CI: 0.82–6.36, *P* = 0.08. MICA-A9 showed no association with a family history of psoriasis or with patient's age at onset of the disease. However, the patients with allele 384 of the C1_4_4 microsatellite, telomeric to HLA-C, were significantly older at the onset of psoriasis (35.3 ± 12.6 versus 25.1 ± 13.7 years, *P* = 0.01) and of arthritis (42 ± 11.5 versus 33 ± 11.7 years, *P* = 0.01). In the same way as MICA-A9, this microsatellite was not associated with a family history of disease.

The following markers were overexpressed in women with PsA: HLA-B*27 (27.3% versus 7.3%. OR 4.8, 95% CI: 1.5–15.5, *P*_*c*_ = 0.01), HLA-C*06 (56.4% versus 16.4%. OR 6.6, 95% CI: 2.7–16.1, *P*_*c*_ = 0.0001), C*07 (49% versus 25.5%. OR 2.8, 95% CI: 1.3–6.3, *P*_*c*_ = 0.01), TNF-308A (45.5% versus 22%. OR 3.0, 95% CI: 1.3–6.9, *P*_*c*_ = 0.009), and MICA-A9 (60% versus 32.7%. OR 3.1, 95% CI: 1.4–6.7, *P*_*c*_ = 0.004). In turn, in men only the following markers were significantly elevated with respect to the male control population: HLA-C*06 (56.4% versus 18.2%. OR 5.8, 95% CI: 2.4–13.8, *P*_*c*_ = 0.0001), HLA-B*27 (36.4% versus 7.3%. OR 7.3, 95% CI: 2.3–23.2, *P*_*c*_ = 0.0004), and MICA-A9 (60% versus 27.3%. OR 4.0, 95% CI: 1.8–8.9, *P*_*c*_ = 0.001).

In reference to the relationship between the genes regulating disease risk and the differential clinical features, 17 out of 24 women with nail disease were C*06 positive (71%) versus only 6 out of 20 males with nail disease (30%), *P* < 0.01. The psoriasis-arthritis latency period was significantly shorter in MICA-A9 positive men (5.4 ± 6.6 years) than in women with this marker (8.2 ± 6.4 years), *P* < 0.01. Similarly, the psoriasis-arthritis latency period was significantly shorter in men with the 384 allele of C4_1_1 (5.1 ± 6.1 years) than in women with this same microsatellite (8.1 ± 6.1 years), *P* < 0.01.

### 3.4. Distribution of Genetic Markers according to Patient Age at Onset of Psoriasis

There were no significant differences in age at the onset of psoriasis between men and women with early psoriasis and HLA-C*06 positivity (21 ± 8.3 versus 18.1 ± 8.9 years, resp.). However, among the individuals with late psoriasis (over 40 years of age) and HLA-C*06 positivity, the age at onset of psoriasis was significantly younger in men (44.3 ± 3.2 years) than in women (57 ± 4.2 years), *P* < 0.01. Similarly, men with C1_4_4 (384) microsatellite positivity and the development of psoriasis after 40 years of age were significantly younger at disease onset (45.7 ± 3.8 years) than the women with the same marker (53.3 ± 7 years), *P* < 0.01. The women developing psoriasis before 40 years of age (*n* = 40) showed significant elevation of the following markers versus the female control population: HLA-C*06 (65% versus 16.4%. OR 9.5, 95% CI: 3.6–24.9, *P*_*c*_ = 0.0001), HLA-C*07 (50% versus 25.5%. OR 2.9, 95% CI: 1.2–6.9, *P*_*c*_ = 0.02), HLA-*B27 (30% versus 7.3%. OR 5.5, 95% CI: 1.6–18.5, *P*_*c*_ = 0.005), and MICA-A9 (62.5% versus 32.7%. OR 3.4, 95% CI: 1.5–8.0, *P*_*c*_ = 0.006). The women developing psoriasis after 40 years of age (*n* = 15) in turn showed over-expression of the following markers: TNF-308A (66.7% versus 22%. OR 9.0, 95% CI: 2.6–31.2, *P*_*c*_ = 0.004) and HLA-DR17–DRB1*03- (53% versus 18%. OR 5.1, 95% CI: 1.5–12.5, *P*_*c*_ = 0.01).

The men developing psoriasis before 40 years of age (*n* = 45) showed over-expression of the following markers: HLA-C*06 (58% versus 18.2%. OR 6.2, 95% CI: 2.5–15.2, *P*_*c*_ = 0.0001), B*27 (42.2% versus 7.3%. OR 9.3, 95% CI: 2.9–30.2, *P*_*c*_ = 0.001), and MICA-A9 (60% versus 27.3%. OR 4.0, 95% CI: 1.7–9.3, *P*_*c*_ = 0.001). The men developing psoriasis after 40 years of age (*n* = 10) in turn showed a significant increase in the following markers: HLA-C*06 (50% versus 18.2%. OR 4.5, 95% CI: 1.1–18.5, *P*_*c*_ = 0.04), MICB-CA23 (50% versus 5.5%. OR 17.3, 95% CI: 3.2–94.9, *P*_*c*_ = 0.001), C1_4_4 (80% versus 22%. OR 14.3, 95% CI: 2.7–76.7, *P*_*c*_ = 0.0007), and MICA-A9 (60% versus 27.3%. OR 4.0, 95% CI: 1.0–16.2, *P*_*c*_ = 0.05) (Tables 3 and 4).

## 4. Discussion

Spondyloarthritic disorders are generally diagnosed more often in men than in women [17]. In reference to the most representative form of these disorders, ankylosing spondylitis, three male cases are documented for every female case [17]. However, since the introduction of the new ASAS criteria for axial spondyloarthritis, these differences are no longer so apparent [18]. PsA appears to be more frequent in men than in women, particularly in its axial presentations [19]. Nevertheless, little is known of the differential clinical expression of PsA between males and females. The explanation for these differences is not clear, and the participation of multifactorial parameters cannot be discarded. In any case, these clinical expression tendencies in men and women with spondyloarthritis appear to persist in the more recently published series [9]. 

We confirmed the greater peripheral involvement in women with PsA (with more frequent polyarthritis), as well as greater physical functional impairment as measured with the HAQ. We do not know whether the same genetic conditioning factors are operative in both males and females with PsA, and the data found in the literature are very limited. Apart from the mentioned relationship between HLA-B27 and the risk of psoriatic spondyloarthritis in males, the only study of note corresponds to that published by Hüffmeier et al., who detected a greater frequency of the R620W mutation of the PTPN22 gene in males with PsA [20].

This study confirmed HLA-C*06, B*27, and MICA-A9 (*002) as three of the most relevant PsA risk genes within the MHC. All three were significantly overexpressed in men and women with PsA. Our data corroborate the growing importance of the PsA risk genes in the MHC. However, these genetic profiles revealed differential characteristics between men and women. Thus, women with psoriatic nail disease were seen to express the C*06 allele significantly more often than men with the same disease. This finding contrasts with the data published by Gudjonsson et al., who reported greater nail involvement in their HLA-C*06 negative psoriatic patients [21]. These same authors found C*06 positive women to be younger at the time of onset of psoriasis than C*06 positive men—an observation not recorded in our series [21]. In fact, among C*06 positive men with an age of over 40 years at onset of the disease, onset proved significantly earlier than in C*06 positive women with an age at onset of psoriasis of over 40 years. In turn, the HLA-B*27 positivity was a marker of early onset disease in both sexes confirming recent observations [22]. 

Patient age at the onset of psoriasis has been a key stratification element in studies of psoriasis. In this sense, it has been known for decades that early psoriasis (manifesting before 40 years of age) is associated with a family history of disease, increased severity, and a stronger correlation to HLA-C*06 positivity, while late psoriasis (manifesting after 40 years of age) appears to be milder, with no clear genetic correlations [23, 24]. Our findings coincide with these observations. However, in our series the impact of the C*06 allele upon disease risk was found to decrease more clearly with age among females (only 33% of the women with psoriasis onset above 40 years of age proved positive for this allele) than in men (50% positivity for this allele in individuals with late psoriasis onset). On the other hand, allele 384 of the C4_1_1 microsatellite was associated with late disease in men but not in women—though this allele was in linkage disequilibrium (LD) with C*06 (*λ*_s_: 0.6). We found that certain markers that are overexpressed in women, such as C*07, TNF-308A and HLA-DR17 (DRB1*03), behave differently according to age at the onset of psoriasis. Specifically, C*07 was seen to be elevated in women with early psoriasis, while the latter two markers were significantly overexpressed only in women with late psoriasis. In women there was a certain LD between TNF-308A, and DR17 (*λ*_s_: 0.3). In any case, it is interesting to note that C*07, TNF-308A, and DRB1*03 form part of the extended haplotype 8.1, which also contains an allele recently linked to PsA risk (HLA-B*08); as a result, it is nowadays difficult to establish which of these alleles are the true disease risk markers [25].

The present work has not been designed as a genetic study, since the small number of patients involved clearly implies a lack of the statistical power needed for such studies. Rather, we aimed to determine whether there are genetic distinctions between men and women that could help explain the little known gender differences in the clinical expression of PsA. Overall, our genetic findings are supported by recent data confirming a good number of our observations referred to the susceptibility role of HLA-B*27, C*06, and C*07 as risk alleles in PsA [22, 25]. As a novelty, we have seen that the impact of these genetic risk elements differs according to patient's gender and age at the onset of psoriasis.

## 5. Conclusions

In sum, men and women with PsA show differences in the expression of the disease, and this is probably due in part to a differential overexpression of certain MHC genes between the two genders. We consider patient's gender and age at the onset of psoriasis to be key stratification factors that should be considered in future studies of the associations between MHC genes and the risk of PsA.

## Figures and Tables

**Table 1:** Clinical characteristics of the study population.

Variables	Men	Women	*P* values
	*n*: 55	*n*: 55	
Age (yr)	46.5 ± 11.6	46.4 ± 15.9	NS
Psoriasis onset age (yr)	27.5 ± 10.4	27.0 ± 14.9	NS
Arthritis onset age (yr)	34.2 ± 9.4	35.1 ± 13.1	NS
Psoriasis duration (yr)	19 ± 11	18.2 ± 8.8	NS
Arthritis duration (yr)	13 ± 7.4	12.5 ± 6.7	NS
Psoriasis-arthritis latency (yr)	7 ± 6.4	7.5 ± 5.6	NS
Family history	40%	45.5%	NS
Oligoarthritis	42%	34.5%	NS
Polyarthritis	20%	40%	<0.05
Axial disease	38.2%	23.6%	NS
Nail disease	36.4%	47.3%	NS
DIP disease	29%	34.5%	NS
IBP (ever)	62%	67.3%	NS
Enthesitis	34.5%	25.5%	NS
Dactylitis	31%	31%	NS
HAQ	0.62 ± 0.42	1.10 ± 0.53	<0.01
Swelling joint count	3.4 ± 4.2	5.2 ± 4.6	<0.05
Tender joint count	10.2 ± 3.4	11.0 ± 4.1	NS
Erosive disease	34.5%	41.8%	NS
PASI	5.4 ± 4.5	5.7 ± 3.8	NS
VSG	15 ± 5.3	16 ± 4.7	NS
HLA-C*06	56.4%	56.4%	NS
HLA-B27	36.4%	27.3%	NS

DIP: distal interphalangeal joint. IBP: inflammatory back pain. HAQ: Health Assessment Questionnaire. PASI: Psoriasis Area and Severity Index.

**Table:** (a) Age at psoriasis onset <40

Variable	Men	Women	*P* values
	*n*: 45	*n*: 40	
Age (yr)	46.3 ± 8.5	48.6 ± 14.6	NS
Arthritis onset age (yr)	35.5 ± 6.8	37.6 ± 10.6	NS
Psoriasis onset age (yr)	25 ± 8.2	22 ± 9.9	NS
Psoriasis duration (yr)	16.3 ± 14.1	21 ± 9.2	0.07
Arthritis duration (yr)	10.9 ± 9.5	11 ± 8.9	NS
Psoriasis-arthritis latency (yr)	5.5 ± 7.1	9.3 ± 6.6	<0.01
Family history	44.4%	50%	NS
Oligoarthritis	46.7%	40%	NS
Polyarthritis	17.7%	37.5%	<0.05
Axial disease	33.3%	22.5%	NS
DIP disease	26.7%	40%	NS
Nail disease	37.8%	52.5%	NS
Enthesitis	35.6%	27.5%	NS
Dactylitis	25.5%	35%	NS
Erosive disease	33.3%	40%	NS
HAQ	0.7 ± 4.5	0.8 ± 6.2	NS
SJC	3.3 ± 4.6	4.3 ± 3.2	NS
TJC	8.2 ± .5.2	9.2 ± 5.5	NS
PASI	7.3 ± 3.4	8.1 ± 4.4	NS

**Table:** (b) Age at psoriasis onset >40

Variable	Men	Women	*P* values
	*n*: 10	*n*: 15	
Age (yr)	59.4 ± 8.4	67.1 ± 8.2	0.03
Arthritis onset age (yr)	49.4 ± 3.4	52.5 ± 9.6	NS
Psoriasis onset age (yr)	45.7 ± 3.4	51.2 ± 6.9	<0.05
Psoriasis duration (yr)	13.6 ± 8	15.9 ± 5.3	NS
Arthritis duration (yr)	9.9 ± 5.9	14.5 ± 9	NS
Psoriasis-arthritis latency (yr)	3.7 ± 3.3	3.2 ± 3.6	NS
Family history	10%	13.3%	NS
Oligoarthritis	0%	20%	NS
Polyarthritis	30%	53.3%	NS
Axial disease	70%	33.3%	NS
DIP disease	30%	13.3%	NS
Nail disease	30%	20%	NS
Enthesitis	30%	20%	NS
Dactylitis	30%	20%	NS
Erosive disease	40%	46.7%	NS
HAQ	0.8 ± 5.1	0.9 ± 5.3	NS
SJC	3.2 ± 4.6	4.7 ± 4.4	NS
TJC	10.3 ± 4.1	11.3 ± 5.2	NS
PASI	4.2 ± 2.3	3.8 ± 3.6	NS

DIP: distal interphalangeal joint. HAQ: Health Assessment Questionnaire. SJC: swelling joint count. TJC: tender joint count. PASI: Psoriasis Area and Severity Index.

**Table:** (a)

Marker	PsA females: 55	Female controls: 55
HLA-C*06	31 (56.4%) OR: 6.6 (2.7–16.1), *P*_*c*_ = 0.0001	9 (16.4%)
HLA-C*07	27 (49%) OR: 2.8 (1.3–6.3), *P*_*c*_ = 0.01	14 (25.5%)
TNF-308A	25 (45.5%) OR: 3.0 (1.3–6.9), *P*_*c*_ = 0.009	12 (22%)
MICA-A9	33 (60%) OR: 3.1 (1.4–6.7), *P*_*c*_ = 0.004	18 (32.7%)
HLA-B*27	15 (27.3%) OR: 4.8 (1.5–15.5), *P*_*c*_ = 0.01	4 (7.3%)

**Table:** (b)

Marker	PsA males: 55	Male controls: 55
HLA-C*06	31 (56.4%) OR: 5.8 (2.4–13.8), *P*_*c*_ = 0.0001	10 (18.2%)
MICA-A9	33 (60%) OR: 4.0 (1.8–8.9), *P*_*c*_ = 0.001	15 (27.3%)
HLA-B*27	20 (36.4%) OR: 7.3 (2.3–23.2), *P*_*c*_ = 0.0004	4 (7.3%)

**Table:** (a) PsA females

Marker	Age at onset <40: 40	Age at onset >40: 15	Female controls: 55
HLA-C*06	26 (65%) OR: 9.5 (3.6–24.9), *P*_*c*_ = 0.0001	5 (33%)	9 (16.4%)
HLA-C*07	20 (50%) OR: 2.9 (1.2–6.9), *P*_*c*_ = 0.02	7 (46.7%)	14 (25.5%)
TNF-308A	15 (37.5%)	10 (66.7%) OR: 9.0 (2.6–31.2), *P*_*c*_ = 0.0004	12 (22%)
HLA-DR17	4 (10%)	8 (53%) OR: 5.1 (1.5–17.5), *P*_*c*_ = 0.01	10 (18%)
MICA-A9	25 (62.5%) OR: 3.4 (1.5–8.0), *P*_*c*_ = 0.006	8 (53.3%)	18 (32.7%)
HLA-B*27	12 (30%) OR: 5.5 (1.6–18.5), *P*_*c*_ = 0.005	3 (20%)	4 (7.3%)

**Table:** (b) PsA males

Marker	Age at onset <40: 45	Age at onset >40: 10	Male controls: 55
HLA-C*06	26 (58%) OR: 6.2 (2.5–15.2), *P*_*c*_ = 0.0001	5 (50%) OR: 4.5 (1.1–18.5), *P*_*c*_ = 0.04	10 (18.2%)
MICB-CA23	1 (2.2%)	5 (50%) OR: 17.3 (3.2–94.9), P_c_ = 0.001	3 (5.5%)
C1_4_4 (384)	12 (26.7%)	8 (80%) OR: 14.3 (2.7–76.6), *P*_*c*_ = 0.0007	12 (22%)
MICA-A9	27 (60%) OR: 4 (1.7–9.3), *P*_*c*_ = 0.001	6 (60%) OR: 4.0 (1.0–16.2), *P*_*c*_ = 0.05	15 (27.3%)
HLA-B*27	19 (42.2%) OR: 9.3 (2.9–30.2), *P*_*c*_ = 0.001	1 (10%)	4 (7.3%)
